# Webcam-Based Pain Measurement Using Pupillary Diameter

**DOI:** 10.3390/s26092746

**Published:** 2026-04-29

**Authors:** Natalia Shamantseva, Arseniy Polyakov, Vsevolod Lyakhovetskii, Margarita Bystrova, Ivan Sakun, Sergey Ananyev, Yury Gerasimenko, Tatiana Moshonkina

**Affiliations:** Pavlov Institute of Physiology, Russian Academy of Sciences, 199034 St. Petersburg, Russia; shandibinan@infran.ru (N.S.); lyakhovetskiiva@infran.ru (V.L.);

**Keywords:** video pupillometry, pupillary diameter, pain measurement, neural network, transcutaneous spinal cord stimulation

## Abstract

Pupillometry can be used as a method for monitoring pain. There are experimental conditions under which standard pupillometry equipment cannot be used. Studying the effects of different pulse forms used for transcutaneous spinal cord stimulation (tSCS) is one such task. The aim was to create a system for recording pupil diameter based on a web camera because it can be synchronised with external equipment, which allows the diameter to be recorded simultaneously with other physiological signals. A markerless system for recording and analysing pupil diameter using deep neural networks was developed based on a commercially available web camera. The accuracy of this system was compared with the accuracy of measurements using manual analysis with ImageJ (version 1.54g). For validation, the system was tested in a study of the dependence of tolerance to tSCS on the shape of stimulating pulses, which involved volunteers (n = 12). The results of the developed pupillometry were compared with the pain rating scale traditionally used in such studies. The developed system is accurate in determining the pupil diameter, comparable to human accuracy. The pupillometry results reproduced those obtained using a subjective pain scale. This method was found to be a reliable method for recording nociceptive pupillary responses in electrophysiology.

## 1. Introduction

In the context of the prevalence of transcutaneous spinal cord stimulation (tSCS) for the modulation of spinal neuronal locomotor networks [1] in neurophysiological movement studies in humans and motor rehabilitation, the significance of kilohertz-modulated pulses for this stimulation has been discussed [2]. Studies have shown that tSCS with a carrier frequency of 5–10 kHz results in better tolerance than conventional rectangular pulses during both single-pulse and train stimulation [3,4]. In these studies, a pain rating scale was used to assess the pain experienced during stimulation. Participants ranked their pain on a scale from 0 to 10, where 0 indicated no pain and 10 indicated the most painful sensation imaginable. A comparative functional study of tSCS using kHz-modulated and conventional pulses is therefore highly relevant [2]. In such a study, it is important to synchronise and monitor both the parameters of movements induced by tSCS and the relationship between motor responses and any discomfort or pain associated with the stimulation. The pain rating scale is subjective and not instrumental. It cannot be used to synchronise with stimulation and recording devices.

Pupillary diameter monitoring is a recognised objective method of measuring sympathetic response associated with pain during anaesthesia and analgesia [5]. While conscious perception of pain is absent in unconscious states, the physiological processing of noxious stimuli remains active. This processing is driven by somatosensory nociceptive signals that ascend to the brainstem and activate the locus coeruleus, which induces pupil dilation through sympathetic activation [6]. Consequently, the pupillary dilation reflex is widely used in clinical practice to quantify nociception and guide the administration of analgesics in sedated or anesthetised patients [5,7,8]. For example, it has been used to objectively quantitate pain intensity and opioid response in paediatric patients [8] to evaluate the efficacy of interventional pain procedures such as nerve blocks for headache management [9], to measure endogenous pain inhibition during thermal stimulation in healthy volunteers [10], and to assess supraspinal pain inhibition during painful electrical stimulation [11].

Infrared pupillometers are used in clinical practice and research studies [5,7]. Ultrasound technology is also beginning to be used for this purpose [12]. However, none of the commercially available devices are suited for the study of motor function modulation through tSCS, since pain measurement must be performed during movement and/or in a specific experimental position. Classical studies of tSCS effects on locomotor networks are typically conducted when individuals are lying on their side, with each leg suspended on an independent swing-like support, allowing horizontal-plane movements of the legs [13,14,15]. In this configuration, the legs are unloaded, making the initiation of locomotor movements by tSCS easier compared with other body and leg positions. This setup is preferable for a comparative functional study of spinal stimulation using kHz-modulated and conventional pulses.

Therefore, as pupillary diameter had previously been associated with pain sensation, the aim of this study was to develop and validate a system for recording and analysing it in the aforementioned experimental setup. This system could then be used to monitor the onset and progression of nociceptive responses during movement investigations by the spinal stimulation. It should digitise the pupillary diameter during the movement studies and synchronise with the other devices in the experimental setup. The two tSCS paradigms (kHz-modulated pulses and conventional pulses) were included for validation purposes because they differ in subjective tolerability.

We selected webcam video recording of the eye because we use the optical motion capture technology to record and visualise movements. Usually, the optical motion capture software synchronises devices for muscle activity recording and the stimulator, and it also allows synchronisation of a webcam.

The proposed pupillary recording system is based on video recording of the eye in the visible light range, recognition of pupil boundaries, and a calibration reference within the video frames using machine learning algorithms and reconstruction of pupil diameter from the detected boundaries and the reference. We tested the developed technology through a comparative study examining tolerance to tSCS with both kHz-modulated and conventional pulses.

This study’s main contribution is the development of an accessible system for recording and analysing pupillary diameter during electrophysiological experiments, using a commercially available webcam and deep neural networks.

## 2. Materials and Methods

### 2.1. Subjects

Twelve healthy volunteers (11 males and 1 female) participated in the study, with a mean age of 25 ± 4 years and body mass index of 23.1 ± 1.6 kg/m^2^. Exclusion criteria included any neurological and musculoskeletal disorder, any acute or chronic disease symptoms, medical or surgical procedures, or trauma within four weeks prior to the study, and pregnancy. All participants self-assessed as healthy on the day of the study. Ethical approval was obtained from the Ethics Committee of the Pavlov Institute of Physiology, Russian Academy of Sciences (Protocol No. 25-07, dated 1 August 2025). The study was conducted in accordance with the Declaration of Helsinki (World Medical Association, 2013). Prior to participation, all participants received detailed information about the study objectives and procedures and provided written informed consent.

### 2.2. Protocol

**Figure 1:**
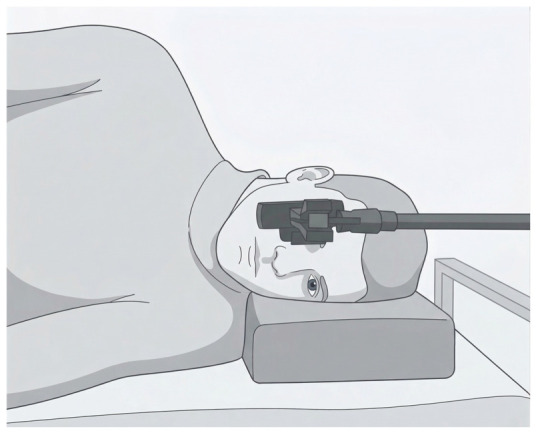
Participants’ positions during the experimental protocol. The webcam is positioned in front of the face to record right pupil dynamics.

Participants were examined in a lateral position on their left side on an examination couch. Both lower limbs were placed on independent horizontal suspension supports, each attached to the ceiling with flexible cables in a swing-like configuration, which allowed for stepping-like movements [13,14]. For each participant, two recording sessions were conducted on the same day. During sessions, participants were instructed to look at the webcam lens positioned in front of their face (Figure 1). Each session began with a 30 s baseline period during which no tSCS was delivered, and participants were instructed to stay relaxed. This baseline served as a control segment for subsequent analysis of pupil area dynamics. Following the baseline period, the spinal stimulation started.

### 2.3. Eye Video Recording

A USB webcam (Logitech C922 Pro Stream, Logitech, Lausanne, Switzerland) was mounted on a microphone stand and positioned approximately 10–15 cm from the anterior surface of the right eye, perpendicular to the pupil plane. Video acquisition was performed at 30 frames per second throughout all experimental sessions. Illumination was provided by a Ulanzi VL49 RGB light (Ulanzi, Shenzhen, China) directed toward the right eye of the participant. The colour temperature was set to 2500 K to maintain consistent warm illumination and minimise spectral variations across sessions. Luminance intensity was monitored using a calibrated lux meter (RADEX LUPIN, RADEX, St. Petersburg, Russia). The illuminance was maintained at 20–40 lux throughout all recordings. Illuminance measurements were performed immediately before each recording session by positioning the lux meter sensor directly adjacent to the lateral canthus of the eye, ensuring that light from the lamp struck the sensor perpendicularly. The illuminance varied by less than 4 lux during testing for each participant.

To account for variations in camera scale caused by head movements or camera displacement during the experiment, a reference was affixed to the skin adjacent to the eye (1–2 cm below the lower eyelid). The reference consisted of a white paper sticker with two parallel red line segments oriented approximately horizontally, with its plane aligned to the anterior ocular surface.

### 2.4. Transcutaneous Spinal Cord Stimulation

Stimulation was delivered using constant-current stimulator Neostim-5 (LTD Cosyma, Moscow, Russia). The cathode (diameter 2.5 cm, ValuTrode^®^ Axelgaard Manufacturing Co., Fallbrook, CA, USA) was positioned midline between the spinous processes of the T11–T12 vertebrae. Two anodes (5 × 10 cm^2^, ValuTrode^®^ Axelgaard Manufacturing Co., Fallbrook, CA, USA) were placed symmetrically over the iliac crests.

Two stimulation paradigms were tested: (1) with single rectangular monopolar pulses not modulated at kHz frequency (MN) and (2) with single rectangular monopolar pulses modulated at a 5 kHz frequency (MM). The duration of the MN and MM pulses was 1 msec. The pulses followed one after another with an interval of about 3 s or longer, and their intensity increased. At each intensity level, three pulses were delivered. This is a procedure for motor evoked potential recording during spinal stimulation that also repeats the protocol from the study of tSCS tolerance that was used in the early research [3,4]. Each stimulation paradigm lasted up to 6 min. Stimulation intensity began at 10 mA and was increased in 10 mA incremental steps until either the participant requested termination or a maximum current intensity of 250 mA was reached. Participants were instructed to verbally indicate when they reached their maximum tolerable intensity (i.e., when they could not tolerate further stimulation). At this point, they reported their perceived pain level using a Visual Analogue Scale (VAS) ranging from 0 to 10, where 0 represented “no pain or discomfort at all” and 10 represented “the worst pain” [16]. The order of the MN and MM paradigms differed among participants, with an interval of at least five minutes between paradigms. The Neostim-5 and webcam were synchronised by Qualisys motion capture system (Qualisys, Gothenburg, Sweden).

### 2.5. Pupil Boundary Detection with DeepLabCut

#### 2.5.1. Training Dataset Preparation

Pupil boundary keypoints were detected using DeepLabCut (version 3.0.0rc9), an open-source deep learning framework for markerless pose estimation [17]. A single-animal project was created.

**Figure 2:**
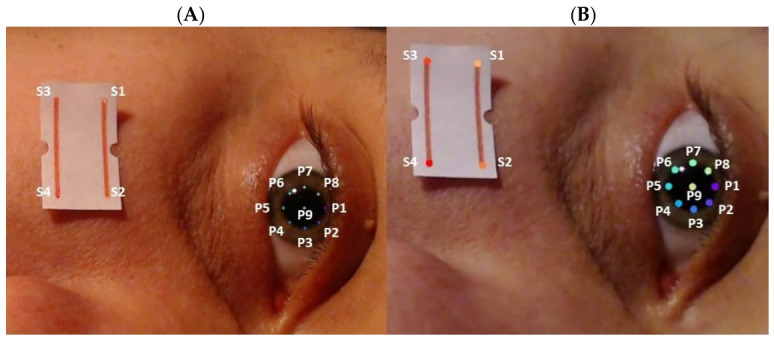
Comparison of manually annotated and neural network-predicted keypoints. (**A**) manually arranged keypoints. (**B**) keypoints arranged by neural network. P1–P8 denote the pupil boundary, P9 indicates the pupil centre, and S1–S4 mark the endpoints of the reference marker (red lines).

Nine keypoints were manually annotated to define the pupil boundary: eight points distributed at 45° intervals around the pupil perimeter (P1–P8) and one at the pupil centre (P9), which was not used in further analysis. Additionally, four keypoints were labelled on a reference visible in the video frame (S1–S4) to serve for subsequent normalisation (Figure 2). The distance between S2 and S4 was 8 mm.

Training frames were extracted from video recordings with k-means clustering to ensure representation of diverse pupil states and lighting conditions. Compared with standard uniform temporal sampling, which risks extracting many redundant frames during static periods, k-means clustering groups frames based on pixel vectors and extracts representatives from distinct clusters [17].

A total of 701 frames (consisting of 92 images from an open dataset and frames extracted across 12 participants) were manually labelled using Napari-DeepLabCut graphical interface. Label placement was performed manually by a single rater based on visual inspection. Label P1 was placed at the uppermost point along the pupil boundary. Labels P2–P8 were subsequently placed in a clockwise direction along the pupil contour. Label P9 was placed at the centre of the pupil. Approximately equal spacing was maintained between all boundary labels. Frames in which keypoints were occluded or not visible were left unlabelled, following established best practices.

Following initial labelling, the training dataset was created with a training fraction of 0.95 (95% training, 5% validation) and shuffle number 1. The dataset was configured with uniform cropping and image augmentation parameters, including affine transformations (rotation ±30°, scaling 0.5–1.25), Gaussian noise (σ = 12.75), and motion blur to enhance model robustness.

#### 2.5.2. Network Architecture and Training

The neural network backbone was a ResNet-50 architecture pretrained on ImageNet, leveraging transfer learning to improve generalisation performance with limited training data. The network consisted of the ResNet-50 feature extractor followed by custom deconvolutional layers to predict heatmaps for keypoint localisation and location refinement fields for sub-pixel accuracy.

Training was performed using the PyTorch (version 2.8.0.) engine in Google Colab with the following hyperparameters: batch size of 8200 training epochs (with model snapshots saved every 5 epochs), AdamW optimizer with initial learning rate of 0.0005, and a learning rate schedule with step decay at epochs 90 and 120 to 0.0001 and 0.00001, respectively [18]. Image crops of 448 × 448 pixels were randomly sampled during training with a maximum shift of 10% to improve spatial invariance. The colour mode was set to RGB, and images were normalised prior to network input. The key metric to choose the best training epoch was the highest value of test Mean Average Precision.

#### 2.5.3. Model Evaluation and Refinement

The model performance was evaluated by the built-in DeepLabCut algorithm after every training session. This algorithm found the average root mean square error between the manual (test dataset) and predicted labels [19]. The pCutoff likelihood filter for the predicted labels was set to 0.2.

Early versions of the model were not accurate enough for the pupil border detection task. To optimise detection accuracy, we labelled more variable frames and added them to the training and validation datasets. Furthermore, an iterative active refinement strategy was employed. During this process, frames with visibly incorrect marker placements were manually identified and relabelled. These corrected frames were then merged into the training dataset, and the network was retrained.

#### 2.5.4. Video Analysis

Following training, the network was applied to all experimental videos to predict keypoint coordinates for every frame. For each detected keypoint, the software outputted x- and y-pixel coordinates alongside a likelihood score (range 0–1) indicating prediction confidence. Results were exported as CSV files containing frame-by-frame coordinates and likelihood values. Additionally, labelled videos with overlaid keypoint predictions were generated to enable visual quality control of the tracking results.

### 2.6. Post-Processing Pipeline in MATLAB

All post-processing analyses were performed in MATLAB (R2023a, MathWorks, Natick, MA, USA). The pipeline consisted of four sequential stages: (1) ellipse fitting to the detected pupil boundary keypoints and area calculation, (2) outlier detection and temporal interpolation, (3) the reference length extraction, and (4) normalisation of pupil metrics by the reference.

#### 2.6.1. Pupil Area Calculation and Ellipse Fitting

The DeepLabCut-generated CSV files containing coordinates and likelihood scores for the nine pupil keypoints (P1–P8: boundary markers; P9: centre) were imported using a custom MATLAB script.

**Temporal smoothing.** To minimise marker instability caused by camera or head movements, a sliding median filter (window = 3 frames, step = 1 frame) was applied to the x- and y-coordinates of each keypoint. This approach allowed for robust boundary reconstruction by utilising a larger set of markers across consecutive frames (up to 24 markers for a 3-frame window).

**Ellipse fitting algorithm**. For each video frame, an ellipse was fitted to the eight pupil boundary points using a two-stage robust fitting procedure:

**Initial fit:** A conic section was fitted to all available valid keypoints using the least squares method via singular value decomposition, which solves the general conic equation: ax^2^ + bxy + cy^2^ + dx + ey + f = 0.

**Outlier removal**: The perpendicular distance from each keypoint to the fitted conic was calculated. Outliers were identified using a robust statistical threshold: points lying beyond median (distance) + k × interquartile range (IQR) were excluded. Additionally, an absolute distance threshold of α × a_0_ was applied, where a_0_ is the semi-major axis length from the initial fit and α = 0.1.

**Refined fit:** The ellipse was re-fitted using only the inlier keypoints. The fitted conic parameters were converted to geometric ellipse parameters (centre coordinates xc, yc; semi-major axis a; semi-minor axis b; orientation angle φ).

**Pupil area.** For all frames where a valid ellipse was fitted (requiring a minimum of 5 keypoints), the pupil area was calculated using the derived semi-axes as A = πab.

#### 2.6.2. Outlier Detection and Temporal Interpolation

To remove physiologically implausible values resulting from tracking errors or blinks, an IQR-based outlier detection algorithm was applied to the pupil area time series. Area values falling outside the range [Q_1_ − 1.5 × IQR, Q_3_ + 3.0 × IQR] were replaced with NaN. Missing values (from outlier removal, low-likelihood frames, or failed ellipse fits) were interpolated using linear interpolation with a maximum gap threshold of 30 frames (~1 s). Gaps exceeding this duration were left as NaN, as longer interruptions typically indicated sustained blinks or pupil occlusions that could not be reliably interpolated.

#### 2.6.3. Reference Length Calculation

To normalise pupil area across participants and account for inter-individual variability in camera-to-eye distance, a visible reference marker was used. The reference consisted of a red line segment captured within the video frame, defined by two endpoints (keypoints S1 and S2 for one endpoint pair, S3 and S4 for another pair).

Reference coordinates were extracted from the DeepLabCut CSV output using a separate MATLAB script. The Euclidean distance L between the most stable marker end-points was calculated frame-by-frame as:

$$L=\sqrt{{\left(Xi-Xj\right)}^{2}+{\left(Yi-Yj\right)}^{2}}$$

where (Xᵢ, Yᵢ) and (Xⱼ, Yⱼ) are the pixel coordinates of the two endpoints.

Normalising reference lengths were processed by: (1) excluding values outside manually set minimum and maximum thresholds; (2) removing statistical outliers using IQR range; and (3) applying linear interpolation to data gaps shorter than a specified duration threshold.

#### 2.6.4. Normalisation Procedure

Pupil area was normalised by the reference length using a custom MATLAB (version R2023a) script to yield dimensionless metrics invariant to camera positioning:

$$Anorm=\frac{A}{L^{2}}$$

where A is pupil area (px^2^) and L is the reference length (px).

Normalisation was performed only for frames in which both the pupil metric and the reference length were available (non-NaN) and the reference length exceeded a minimum threshold of 1 × 10^−6^ pixels to avoid division by near-zero values. The normalised time series were saved for subsequent statistical analysis.

### 2.7. Validation of Neural Network Predictions

To validate the accuracy of pupil area measurements obtained from the neural network, manual measurements were performed in ImageJ (version 1.54g) (National Institutes of Health, Bethesda, MD, USA) using ellipse fitting on a subset of frames. 120 frames were randomly selected from the first 30 s (baseline period) of the initial recording session across all 12 participants (10 frames per participant). For each frame, three measurements were independently calculated using both ImageJ manual analysis and the neural network: (1) pupil area in pixels, (2) distance between the two most stable reference keypoints (S2 and S4) in pixels, and (3) pupil diameter converted to millimetres using the calibrated reference length (8 mm).

Normality of the data was assessed using the Shapiro–Wilk test. Since the data did not follow a normal distribution, agreement between manual and automated measurements was evaluated using Bland–Altman analysis [20] for repeated measures with non-parametric summary statistics. Bias was expressed as the median difference with interquartile range [Q1; Q3], and limits of agreement (LOA) were calculated as the 2.5th and 97.5th percentiles of the differences. Additionally, Spearman correlation coefficients for repeated measurements were computed to assess the strength of linear association between the two methods. All statistical analyses were performed in RStudio (version 4.3.1, R Foundation for Statistical Computing, Vienna, Austria).

### 2.8. Analysis of the Relationship Between Stimulation Intensity, Pain, and Pupil Diameter

For each tSCS paradigm (MM and MN), two time intervals were extracted from the continuous tracking data for comparison: (1) a 30 s baseline period (900 frames) recorded prior to the stimulation, and (2) a 9 s period (270 frames) corresponding to the maximum tolerable stimulation intensity. Three participants who tolerated the maximum stimulation intensity of 250 mA without requesting termination were excluded from this analysis, as their data did not contain a clearly defined tolerance threshold. The remaining nine participants were included in the statistical comparison. No likelihood filtering was applied to the tracking data.

To process the extracted frames, the normalised pupil area (A) was averaged across all frames within each respective time interval. This averaged normalised area was then converted into an absolute pupil diameter (D, in mm) using the formula D = 8 × 2√(A/π), where 8 represents the physical reference distance (in mm) between the S4 and S2 markers.

The pupillary response, defined as the absolute change in pupil diameter (in mm), was calculated for each stimulation paradigm by subtracting the average baseline diameter from the average diameter at the maximum tolerable intensity.

Since the data was non-normally distributed, the Wilcoxon signed-rank test was used to evaluate whether pupil diameter during stimulation differed significantly from baseline. A *p*-value < 0.05 was considered statistically significant.

## 3. Results

### 3.1. Network Performance Metrics

The final training session consisted of 186 epochs. At the 180th training epoch, the ResNet-50-based neural network achieved robust detection performance on the validation set. Quantitative evaluation metrics are summarised in Table 1.

The network achieved Mean Average Precision (mAP) of 87.54% and Mean Average Recall (mAR) of 89.44% on the validation set, indicating high accuracy in keypoint prediction. The RMSE was 8.14 pixels for the test set.

### 3.2. Agreement with ImageJ Manual Calculations

**Figure 3:**
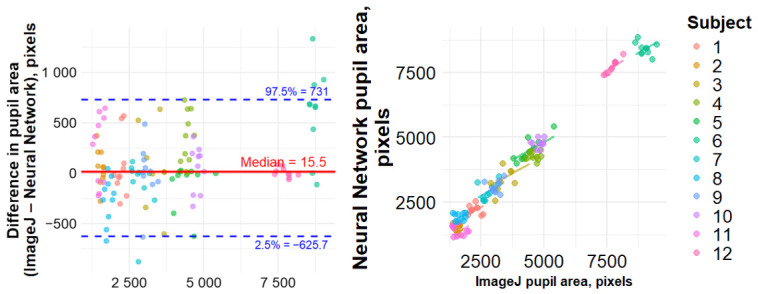
Results of non-parametric Bland–Altman (**left**) and Spearman’s correlation (**right**) analyses for paired measurements comparing pupil area (square pixels) obtained using the neural network and ImageJ.

A Bland–Altman analysis comparing the pupil area determined by a neural network and manual measurements demonstrated a median bias of 15.5 [−83.1; 200.3] square pixels and limits of agreement (LOA) of −626 and 731 square pixels. The two methods were moderately correlated (r = 0.51, *p* < 0.001) (Figure 3).

**Figure 4:**
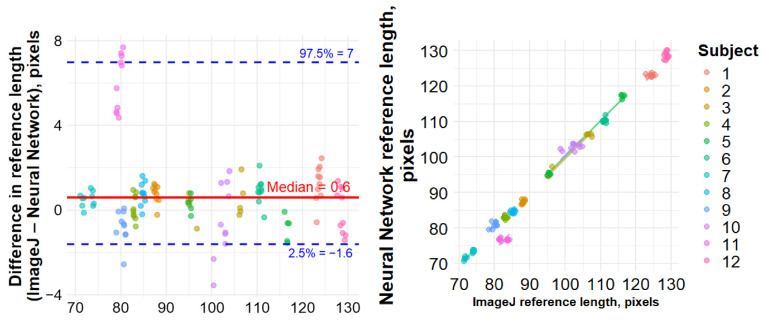
Results of non-parametric Bland–Altman (**left**) and Spearman’s correlation (**right**) analyses for paired measurements comparing reference length (pixels) obtained using the neural network and ImageJ.

The bias in a reference length (S4 − S2) was 0.6 [−0.2; 1.1] pixels, with LOA from −1.6 to 6.9 pixels and a correlation of 0.97 (*p* < 0.001) (Figure 4).

**Figure 5:**
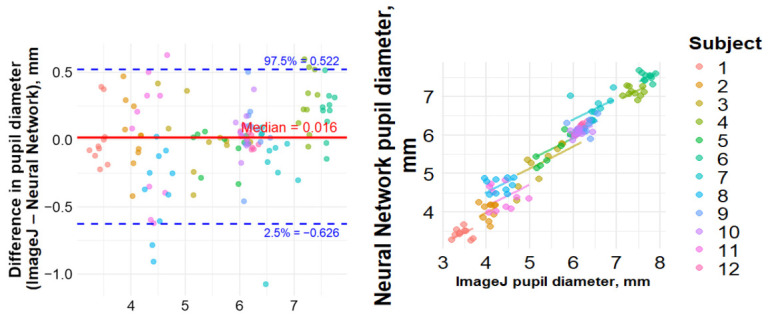
Results of non-parametric Bland–Altman (**left**) and Spearman’s correlation (**right**) analyses for paired measurements comparing pupil diameter (mm) obtained using the neural network and ImageJ.

The bias in pupil diameter was 0.016 [−0.08; 0.176] mm, with LOA from −0.62 to 0.52 mm and a correlation of 0.53 (*p* < 0.001) (Figure 5).

### 3.3. Maximum Tolerable Intensity

**Figure 6:**
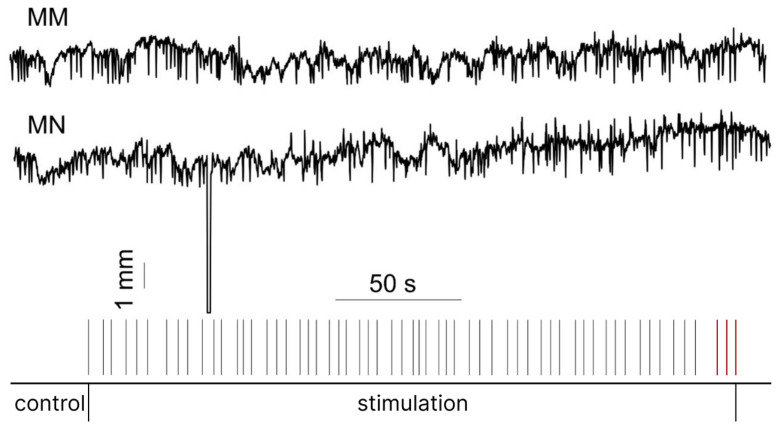
Individual data showing the syncronisation between stimulation pulses and pupillary diameter (mm). The first 30 s of each recording served as a control. At each intensity level, three pulses were delivered. Red lines indicate the maximum tolerable intensity. MM—monopolar modulated, MN—monopolar non-modulated.

Maximum tolerable intensities were 240 [215; 250] mA for MM and 235 [120; 240] mA for MN pulses. Maximum tolerable intensity for the MM pulses tended to be higher than for the MN pulse (*p* = 0.06). Figure 6 illustrates the time course of pupil diameter aligned with stimulation onset for the MN and MM paradigms (Figure 6).

Table 2 shows individual maximum tolerable intensity values and the corresponding VAS scores (Table 2).

### 3.4. Pupil Diameter Versus Stimulation Paradigm

The pupil diameter before the MM paradigm was 5.9 [4.3; 6.2] mm, and before the MN paradigm was 5.7 [4.3; 6.1] mm. No significant differences were obtained between these control values (*p* > 0.05).

**Figure 7:**
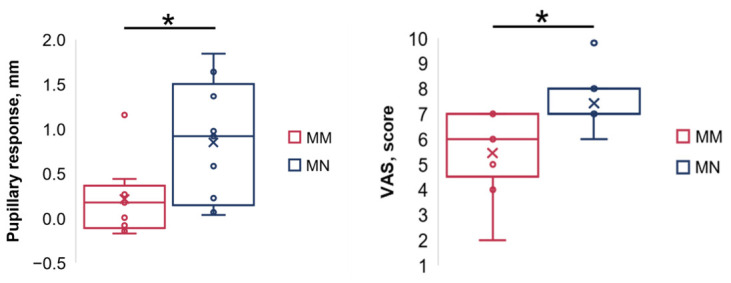
Pupillary responses (**left**) and Visual Analogue Scale scores (**right**) for the monopolar modulated (MM) and monopolar non-modulated (MN) stimulation paradigm; number of participants = 9. Box plots show the median (horizontal line), interquartile range (box), and minimum–maximum range (whiskers); individual data points are shown as circles; the cross (×) denotes the mean. * *p* < 0.05.

At the maximum tolerable intensity, the pupil diameter was 6.2 [4.3; 6.6] mm for the MM paradigm, with a pupillary response of 0.2 [−0.1; 0.3] mm, which was not significantly different compared with the control value (Figure 7). The MN paradigm was found to significantly dilate the pupil by up to 6.4 [5.4; 7.1] mm (*p* = 0.03), producing a pupillary response of 0.9 [0.2; 1.4] mm. The pupillary response for the MN paradigm was significantly greater than for the MM paradigm (*p* = 0.01).

### 3.5. Visual Analogue Scale Score Versus Stimulation Paradigm

VAS scores were significantly higher for the MN pulses and were equal to 7 [7; 8], compared with 6 [4; 7] for the MM pulses (*p* < 0.01) (Figure 7).

## 4. Discussion

This study shows that webcam-based video pupillometry combined with DeepLabCut provides a practical and sensitive approach for monitoring pupillary diameter during noninvasive spinal stimulation. The approach enabled continuous, synchronised assessment of pupil dynamics and demonstrated that pupil diameter differentiates between tSCS paradigms at maximum tolerable intensity.

### 4.1. Comparison with Existing Methods

The comparison of the proposed method with existing technologies and algorithms for pupil diameter assessment is provided in Table S1 in the Supplementary Materials.

In contrast to infrared pupillometers and ultrasound-based approaches, which are often limited to static clinical settings [5,7,12], the proposed method, operating with a standard RGB webcam and flexible mounting, makes it compatible with a side-lying, leg-suspended locomotor setup [13,14,15]. While clinical devices typically provide standardised optics and illumination, they are less suited for dynamic tSCS protocols requiring integration with a motion capture system and electromyography. In this study, the use of DeepLabCut for markerless pupil boundary detection and reference lines allowed for the reconstruction of pupil diameter in millimetres under a common spinal stimulation protocol [3,4,14].

Several deep learning approaches for pupil boundary detection have been proposed [21,22,23,24]. Convolutional neural networks for visible-light pupillometry have been developed to estimate elliptical pupil parameters directly from images, eliminating the need for a separate ellipse-fitting step. DeepLabCut has been successfully applied to detect pupil boundaries in rodent eye-tracking studies, and dedicated repositories provide pre-trained models for rodent pupillometry. The use of Raspberry Pi computers and cameras is a key component of this technique, which “require[s] extensive technical and experimental expertise and may not be available to the majority of laboratories” [21]. Other work describes an algorithm for obtaining a pupil mask and extracting the parameters of an ellipse that represents the boundary of an elliptical pupil recorded in visible light [22]. This algorithm has a limitation in that it uses a training dataset containing images that differ from the experimentally acquired images. The experimental images were captured with a commercially available Android smartphone. A webcam-based deep learning eye-tracking system has also been explored for online behavioural experiments [23]. This system records eye fixations, eye movements, and blinks, but not pupil diameter. However, none of these implementations address the specific requirements of our experimental paradigm.

Traditional computer vision methods for pupil detection, such as the circular Hough transform, ellipse fitting, Daugman’s integro-differential operator, and the radial symmetry transform, rely on handcrafted features and perform well under controlled infrared illumination with minimal occlusion [25]. However, these algorithms are sensitive to specular reflections, partial eyelid occlusion, and motion blur, which are unavoidable in a visible-light setup where stimulation-evoked movements shift the head and face within the camera field of view. In this study, the use of DeepLabCut’s ResNet-50 backbone with transfer learning and iterative active refinement allowed robust keypoint detection in visible light across diverse pupil states, lighting conditions, and head positions, achieving a mAP of 87.54% and mAR of 89.44% on the validation set despite the challenging recording environment [17].

### 4.2. Pupillary Response to Transcutaneous Spinal Cord Stimulation

In our study, the MN stimulation paradigm was less tolerable than the MM paradigm. Three results demonstrate this effect: the tolerable intensity was lower in the MN paradigm than in the MM paradigm, the VAS score was higher in the MN paradigm, and the pupil diameter and its change were higher in the MN paradigm. The significant pupillary dilation of 0.9 mm observed during the MN paradigm aligns with the established physiological ranges reported in conscious adults, where noxious somatic or electrical stimulation evokes a reflexive pupil dilation of 0.4 ± 0.2 mm [26,27]. These results are consistent with previous studies that found tolerance to kHz-modulated pulses was higher than tolerance to conventional pulses [3,4,28]. In previous studies, researchers tested tolerance to tSCS using a pain rating scale or stimulation until participants reported it was too intense.

### 4.3. Future Directions

Taken together, our results demonstrate that the developed system for recording and analysing pupil diameter can be used in further studies of the functional effects of tSCS with conventional and modulated pulses and the relationship between functional effect and tolerance to stimulation. In such experiments, tSCS is delivered continuously at 15–30 Hz for several minutes while the participant lies in the side-lying position with legs suspended, producing rhythmic stepping movements [14]. Because the stimulation is ongoing and movements are continuous, pain and discomfort cannot be assessed using single-point subjective scales; instead, a dynamic objective measure is required. The webcam-based pupillometry system presented here meets this requirement by providing frame-by-frame pupil diameter values that are synchronised with electromyography and motion capture data. Future work will extend the current single-pulse protocol to continuous tSCS paradigms and will evaluate whether differences in pupillary responses across waveforms observed during singl-pulse stimulation are replicated during sustained locomotor activity.

## 5. Limitations

The relatively wide limits of agreement suggest that the system’s sensitivity to more subtle pupillary changes, such as those seen in the MM condition, remains limited. Consequently, the proposed method is adequate for the coarse monitoring of stimulation-evoked responses and is best suited for detecting larger group-level changes within this specific experimental setup.

We opted not to apply likelihood-based filtering, as the combination of long recording sessions (up to 6 min) and stimulation-induced movement artifacts made standard thresholds impractical. Tracking quality was therefore verified through visual inspection.

Another limitation of the method is the difficulty of controlling illumination during video acquisition. When the optical contrast between the pupil and the iris is low, a high light intensity is required. Additionally, controlled (directional) illumination is necessary because diffuse or irregular lighting produces multiple reflections that obscure the pupil-iris boundary.

Another limitation of the method is the necessity of a reference that compensates only for uniform changes in imaging scale, specifically situations in which the pupil moves closer to or farther from the camera. In cases of non-uniform scale changes, such as rotation of the plane containing both the pupil and the reference (e.g., due to head tilt), normalisation would require a two-dimensional reference object with a known area (e.g., a triangle, square, or ellipse), rather than a line segment. Accordingly, the DeepLabCut model would need additional training to detect at least three points of the normalisation marker to reconstruct the triangular boundary and compute its area.

The final limitation of the method is that the current model is only trained to recognise a specific reference. If a researcher uses a different object as a reference, the model will require additional training.

## 6. Conclusions

We have developed a system for recording and analysing pupillary diameter in a human movement study protocol to monitor nociceptive responses during spinal stimulation. The system uses a webcam and works in standard room lighting. It is synchronised with electrophysiological equipment. It can digitise pupillary diameter during movement experiments.

The developed approach made it possible to objectively distinguish differences in tolerance between stimulation paradigms. In particular, the findings showed that kilohertz-modulated stimulation was associated with lower pupillary response and better tolerance than conventional stimulation. These results validate the proposed synchronised system, confirming its ability to reliably detect and monitor differences in pupillary responses during electrophysiological studies involving spinal stimulation.

## Figures and Tables

**Table 1:** DeepLabCut neural network performance metrics on training and validation datasets.

Metric	Training Set	Validation Set
Mean Average Precision (mAP), %	90.81	87.54
Mean Average Recall (mAR), %	92.76	89.44
Root Mean Squared Error (RMSE), pixels	7.34	8.14
RMSE at pCutoff = 0.2, pixels	6.77	7.42

**Table 2:** Individual maximum tolerable intensity (mA) and visual analogue scale (VAS) scores for non-modulated (MN) and 5 kHz-modulated (MM) transcutaneous spinal cord stimulation paradigms.

Participant	MN		MM	
	Current, mA	VAS	Current, mA	VAS
1	240	7	240	7
2	235	8	230	5
3	240	6	240	4
4	200	7	200	5
5	90	7	130	6
6	240	8	250	7
7	240	7	250	2
8	140	9	240	7
9	100	7	250	6

## Data Availability

The data supporting the findings of this study are available upon request from the corresponding author.

## Supplementary Materials

The following supporting information can be downloaded at: https://www.mdpi.com/article/10.3390/s26092746/s1. Table S1: Comparison of the proposed method with existing technologies and algorithms for pupil diameter assessment.
