# Supplementary material for: Webcam-Based Pain Measurement Using Pupillary Diameter
Source: Sensors (Basel). 2026 Apr 29;26(9):2746. doi: 10.3390/s26092746 (PMC13165936; doi:10.3390/s26092746)
Supplement: Supplementary file 1 [file sensors-26-02746-s001.zip › sensors-4214265-supplementary.pdf]

Table S1. Comparison of the proposed method with existing technologies and algorithms for pupil diameter assessment

Technology / Algorithm	References	Key Advantages	Primary Limitations
Infrared Clinical Pupillometers	[1–3]	Clinical gold standard; high precision; automated algorithms eliminate human error.	Bulky or handheld; restricted to static clinical settings; cannot synchronise with external motion capture.
Ultrasound Pupillometry	[4,5]	Can be used when the eyelid is closed or swollen (e.g., severe trauma).	Requires physical contact (gel/probe) on the eyelid; unsuitable for awake, moving subjects.
Commercial Screen-Based Eye-Trackers	[6]	Non-contact; suitable for screen-based cognitive tasks; high sampling rate.	Locked to a monitor; proprietary software makes it difficult or impossible to synchronise with external hardware (EMG, tSCS).
Smartphone Applications	[7,8]	Accessible hardware; non-invasive; low cost.	Standardised apps cannot output real-time analog/digital triggers to sync with electrophysiological equipment.
Traditional Computer Vision Algorithms	[9]	Computationally lightweight and fast; accurate for perfectly circular pupils under controlled infrared illumination.	Sensitive to specular reflections, motion blur, and low contrast in visible light; prone to failure when the pupil is elliptical or partially occluded.
Proposed Method (RGB Webcam + DeepLabCut)		Markerless; low-cost; robust to partial occlusions and visible-light reflections; designed to synchronise with EMG/motion capture.	Requires manual calibration of a physical reference marker; computationally heavy model training required.

References

1. Packiasabapathy, S.; Rangasamy, V.; Sadhasivam, S. Pupillometry in Perioperative Medicine: A Narrative Review. *Can J Anaesth* **2021**, *68*, 566–578, doi:10.1007/s12630-020-01905-z.
2. Ledowski, T. Objective Monitoring of Nociception: A Review of Current Commercial Solutions. *Br J Anaesth* **2019**, *123*, e312–e321, doi:10.1016/j.bja.2019.03.024.

3. Phillips, S.S.; Mueller, C.M.; Nogueira, R.G.; Khalifa, Y.M. A Systematic Review Assessing the Current State of Automated Pupillometry in the NeuroICU. *Neurocrit Care* **2019**, *31*, 142–161, doi:10.1007/s12028-018-0645-2.
4. Fratino, S.; Garré, A.; Garufi, A.; Hafidi, S.; Migliorino, E.; Stropeni, S.; Bogossian, E.G.; Ndieugnou Djangang, N.; Albano, G.; Creteur, J.; et al. Evaluation of Nociception in Unconscious Critically Ill Patients Using a Multimodal Approach. *Anaesth Crit Care Pain Med* **2023**, *42*, 101175, doi:10.1016/j.accpm.2022.101175.
5. Rajendran, G.; Mahalingam, S.; Ramkumar, A.; Krishnamoorthy, Y.; Kumaresh, P.T.; Vijayan, V.; Elanjaeran, R.; Kannan, R.; Prakasam, S.; Salih, A. Ultrasound for Pupillary Assessment - A Systematic Review. *JEM Reports* **2025**, *4*, 100158, doi:10.1016/j.jemrpt.2025.100158.
6. Fernandez Rojas, R.; Brown, N.; Waddington, G.; Goecke, R. A Systematic Review of Neurophysiological Sensing for the Assessment of Acute Pain. *npj Digital Medicine* **2023**, *6*, 76, doi:10.1038/s41746-023-00810-1.
7. Szabadi, E. Modulation of Physiological Reflexes by Pain: Role of the Locus Coeruleus. *Front Integr Neurosci* **2012**, *6*, 94, doi:10.3389/fnint.2012.00094.
8. Shekhar, V.; Choudhary, N.; Rathore, P.; Singh, S.P.; Bhatnagar, S. Non-Invasive Objective Markers to Measure Pain: A Direction to Develop a Pain Device - A Narrative Review. *Indian J Palliat Care* **2023**, *29*, 217–222, doi:10.25259/IJPC_257_2022.
9. Romaguera, T.V.; Romaguera, L.V.; Piñol, D.C.; Seisdedos, C.R.V. Pupil Center Detection Approaches: A Comparative Analysis. *Computacion Y Sistemas* **2021**, *25*, 67–81, doi:10.13053/cys-25-1-3385.
